# *Candida glabrata* species complex in a Brazilian public tertiary hospital: site of infection, identification and antifungal susceptibility profile

**DOI:** 10.1590/1678-9199-JVATITD-2025-0098

**Published:** 2026-08-10

**Authors:** Maricelma Francelino Fialho Cândido, Francine de Sales Dorneles, Juliana Possatto Fernandes Takahashi, Beatriz Aparecida Soares Pereira, Camila Marçon, Dálity Keffelen de Barros Rodrigues, Lídia Raquel de Carvalho, Marcia de Souza Carvalho Melhem, Anamaria Mello Miranda Paniago, Rinaldo Poncio Mendes

**Affiliations:** 1 Graduate Program on Infectious and Parasitic Diseases, School of Medicine, Federal University of Mato Grosso do Sul (UFMS), Campo Grande, MS, Brazil.; 2 Pathology Division, Adolfo Lutz Institute, Secretariat of Health, São Paulo, SP, Brazil.; 3 Botucatu Medical School, São Paulo State University (Unesp), Botucatu, SP, Brazil; 4 Biosciences Institute, São Paulo State University (Unesp), Botucatu, SP, Brazil.; 5 Laboratory of Medical Mycology LIM-53, Division of Clinical Dermatology, Institute of Tropical Medicine, Hospital das Clínicas, School of Medicine, University of São Paulo (USP), São Paulo, SP, Brazil.

**Keywords:** Candida glabrata, Candidemia, Site of infection, Identification, Antifungal susceptibility

## Abstract

**Background:**

The increasing incidence of *Candida glabrata* complex infections in hospitalized patients and their association with high mortality rates prompted the determination of cryptic species among clinical isolates from a tertiary hospital and the evaluation of their susceptibility profiles to commonly prescribed antifungal agents.

**Methods:**

This study evaluated 80 *C. glabrata* isolates obtained from patients admitted to a Brazilian public tertiary hospital. The isolates were recovered from different clinical specimens, predominantly urine and blood, across various medical units.

**Results:**

MALDI-TOF MS analysis revealed that all isolates were *Candida glabrata sensu stricto*. Minimum inhibitory concentrations (MICs), determined via broth microdilution and according to the European Committee on Antimicrobial Susceptibility Testing (EUCAST) guidelines, showed high susceptibility to amphotericin B (AmB), voriconazole (VRC), and echinocandins (ECNs) [caspofungin (CSF), anidulafungin (ANF), and micafungin (MCF)], as well as uniform susceptibility within the "I" category (susceptible, increased exposure) to fluconazole (FLC). Comparisons of resistance profiles revealed higher prevalences of resistance to AmB and VRC than to ECNs, both overall and in urine isolates, with a similar trend observed in blood isolates. Comparative analysis with reference EUCAST *C. glabrata* data highlighted higher localized MIC values for AmB, ANF, and MCF, lower MICs for FLC, and equivalent distributions for VRC.

**Conclusion:**

All recovered isolates were confirmed as *Candida glabrata sensu stricto* and exhibited different susceptibility profiles from reference EUCAST isolates. These findings establish a strong regional baseline, serving as a useful guide for monitoring therapeutic measures and supporting antimicrobial stewardship.

## Background

Yeasts of the genus *Candida* exhibit several clinically relevant characteristics. Certain species can colonize various human organs, either transiently or for extended periods, including the oral cavity, gastrointestinal tract, vagina, and skin. Their opportunistic behavior, in which pathogenicity is determined by host factors, leads to diverse clinical manifestations depending on the underlying disease. For example, hematogenous dissemination in neutropenic patients can result in hepatosplenic candidiasis, while mucosal involvement in cases of cell-mediated immunosuppression, such as that in HIV-infected patients, can manifest as esophageal candidiasis, an AIDS-defining condition [1].

The escalating resistance of certain *Candida* species to antifungal compounds has become a significant clinical concern [2]. Notably, *Candida glabrata (Nakaseomyces glabrata)* frequently contributes to antifungal resistance in hospitalized patients [3-5]. Despite its inability to undergo the yeast-to-mycelium transition or to secrete proteases, the incidence of *C. glabrata* infections has been rising, particularly among inpatients [3, 6]. This pathogen is associated with a relatively high mortality rate, which is a substantial public health burden [3].

The *Candida glabrata* complex, a group of pathogenic species, comprises *Candida glabrata* sensu stricto, *Candida bracarensis*, and *Candida nivariensis* [7]. However, a study encompassing 1,595 isolates from various countries revealed that only 1 (0.06%) was *C. nivariensis* and 2 (0.13%) were *C. bracarensis* [7]. Conversely, 16 *C. nivariensis* strains, isolated from patients across 12 hospitals in the United Kingdom over a two-year period, exhibited reduced susceptibility to fluconazole (FLC), itraconazole (ITC), and voriconazole (VRC) [8]. These findings highlight the low prevalence of *C. bracarensis* and *C. nivariensis,* while underscoring the reduced susceptibility of *C. nivariensis* to triazole antifungal agents. Notably, studies characterizing species within the *C. glabrata* complex remain scarce [9-14].

Treatment of *C. glabrata* infections involves antifungal compounds from three primary pharmacological classes: amphotericin B deoxycholate (AmBd) and its lipid formulations, triazoles (FLC, VRC), and echinocandins (caspofungin - CSF, anidulafungin - ANF, and micafungin - MCF). AmBd, a polyene antibiotic, exerts its fungicidal effect by binding to ergosterol in the cell membrane, thereby disrupting membrane permeability [15]. Triazoles, acting as fungistatic agents, inhibit 14-α-sterol demethylase, a key enzyme in ergosterol biosynthesis [15]. Echinocandins, polypeptides with fungicidal activity against *Candida* species, inhibit β-(1,3)-D-glucan synthase, which is essential for β-(1,3)-glucan synthesis, a crucial component of the fungal cell wall [15].

Given the increasing incidence of *C. glabrata* infections in hospitalized patients, their observed antifungal resistance, and the associated high mortality rates, the present study aimed to determine the occurrence of cryptic species among clinical isolates from patients at a tertiary hospital and to evaluate their susceptibility profiles to commonly prescribed antifungal agents.

## Methods

### Study setting

This study evaluated 80 *C. glabrata* isolates obtained from patients admitted to the State Hospital of Bauru, a public tertiary hospital located in the central-west region of São Paulo State, Brazil. Patient isolates were collected from various hospital units, including general wards, the emergency room intensive care unit (ICU), the emergency room, the adult ICU, the coronary care unit (CCU), the clinical coronary unit, the burn unit ICU, the chemotherapy unit, the ambulatory medical care clinic, the shelter accommodation unit, and the pediatrics department.

### Study design

This retrospective observational study examined a cohort of patients with *C. glabrata* infections. Urine isolates were considered the etiological agent only when the colony-forming unit count exceeded 100,000/mL. Only one isolate per patient was included. Laboratory evaluations were conducted at the Laboratory of Infectious and Parasitic Diseases, School of Medicine, Federal University of Mato Grosso do Sul.

### Isolates

The 80 clinical isolates were predominantly obtained from urine (67.5%), blood (18.8%), and tracheal secretions (7.5%) (Additional file 1).

### Species identification

Isolate identification was performed using proteomics via matrix-assisted laser desorption/ionization time-of-flight mass spectrometry (MALDI-TOF MS). Prior to extraction with formic acid and acetonitrile, as recommended by the manufacturer (Bruker-Daltonics, Bremen, Germany), isolates were subcultured on CHROMagar Candida® (Difco, USA). The resulting extract was pipetted in duplicate onto 96-spot metal plates. Dried samples were then overlaid with 1 μL of matrix (α-cyano-4-hydroxycinnamic acid, HCCA) and analyzed using Biotyper RTC 3.0 software, based on protein spectrum matching. Results were scored as follows: 2.00-3.000 (highly probable species identification), 2.000-2.299 (confident genus and probable species identification), 1.700-1.999 (probable genus identification), or 0.000-1,699 (unreliable identification).

### Antifungal susceptibility

Antifungal susceptibility testing was conducted using six antifungal agents (Sigma, USA): amphotericin B (AmB), caspofungin (CSF), micafungin (MCF), anidulafungin (ANF), fluconazole (FLC), and voriconazole (VRC). Minimum inhibitory concentrations (MICs) were determined for all *C. glabrata* isolates via broth microdilution, adhering to the European Committee on Antimicrobial Susceptibility Testing (EUCAST) guidelines as outlined in document E.DEF 7.3.2 [16].

Stock solutions were prepared with dimethyl sulfoxide and diluted in Roswell Park Memorial Institute (RPMI) liquid medium (Sigma, USA) to achieve ten final drug concentrations, ranging from 0.0008 mL/L to 4 mL/L, except for FLC, which was tested at 12 concentrations (0.008 mg/L to 32 mg/L). *C. krusei* ATCC 6258 and *C. parapsilosis* ATCC 22019 reference strains were included as internal quality controls in all microdilution assays [17]. Tests were performed at 35°C ± 2°C, and fungal growth inhibition was measured spectrophotometrically at 492 nm after 24 hours of incubation. The MIC was defined as the lowest concentration inhibiting > 90% of fungal growth (IC_90_) for AmB and > 50% inhibition (IC_50_) for azoles and echinocandins, relative to the positive control. MIC_50_ and MIC_90_ parameters were employed to analyze the results, representing the drug concentrations required to inhibit 50% and 90% of the isolates, respectively. Data calculations were performed using Microsoft Office Excel 2007. Clinical breakpoints were applied where available; otherwise, epidemiological cutoff values (ECOFFs) were utilized. Breakpoints and ECOFFs were adopted according to the 2023 EUCAST recommendations [18].

For caspofungin (CSF), since no defined breakpoints were available, the 2020 EUCAST recommendation to consider isolates susceptible to anidulafungin (ANF) as susceptible to CSF was followed [18].

### Sample size required

The sample size was calculated for a study comparing two tests in the same sample, assuming a type I error of 0.05, a type II error of 0.20 (corresponding to a power of 0.80), a ratio between the discrepant data, and a lower ratio of 0.10 between the discordant data. Based on these parameters, the estimated minimum sample size was 48 isolates [19].

### Control group

MIC results for the isolates were compared with those published by EUCAST [20].

### Statistical analysis

Frequencies within the same sample were compared using the one-sample chi-square test [21]. The chi-square test and Fisher’s exact test were used to compare frequencies between independent samples. When more than two frequencies from independent samples were compared, the chi-square test followed by the Goodman post-hoc test was applied. Comparisons of more than two frequencies within the same sample were performed using Cochran’s Q test. Kendall’s coefficient of correlation (concordance) was used to evaluate the correlation between variables. Statistical analyses were performed using SPSS software (version 9.4), except for the Goodman test, which was performed using a program standardized by the Biostatistics Section of the Institute of Biosciences, Unesp, Botucatu, SP, Brazil. All values were expressed as two-sided *p-*values, with *p* ≤ 0.05 taken to define a statistically significant result.

## RESULTS

### Sites of infection and clinical specimens

Most patients with *C. glabrata* infections were admitted to general wards, with similar frequencies of hospitalization across other units (Additional file 2). *C. glabrata* was most frequently isolated from urine (67.50%) and blood (18.75%) samples (Additional file 1).

### Antifungal susceptibility

All isolates underwent evaluation of antifungal activity for the six antifungals. The results are presented in Table 1. None of the *C. glabrata* isolates were susceptible to FLC (MIC < 0.001), 76 showed susceptible, increase exposure (I) susceptibility and four were resistant. The four FLC-resistant isolates were non-wild type for VRC, but three isolates with I FLC susceptibility were non-wild type for VRC. The echinocandins evaluated showed different MIC values, with CSF and MCF values lower than those of ANF. Regarding the triazole derivatives, VRC showed a lower MIC than FLC (Additional file 3). The evaluation of the 80 isolates revealed that the prevalence of resistant or non-wild type strains was higher for AmB than for the echinocandins, with VRC occupying an I position. Similar finding was observed when the 54 urine isolates were evaluated. However, the prevalence of resistant isolates did not differ, according to the antifungal compound evaluated, for blood and tracheal secretion samples (Table 2).

**Table 1.** Distribution of minimum inhibitory concentration (MIC) values for antifungals against 80 clinical isolates of *Candida glabrata* (São Paulo State, 2015-2023).

Antifungal agent	Number of isolates at MIC (mg/L)												
	0.008	0.016	0.03	0.06	0.125	0.25	0.5	1	2	4	8	16	32
Micafungin	5	5	70	0	0	0	0	0	0	0	NE	NE	NE
Anidulafungin	0	0	36	43	0	0	0	1	0	0	NE	NE	NE
Caspofungin*	11	11	56	2	0	0	0	0	0	0	NE	NE	NE
Fluconazole	0	4	0	3	6	8	13	15	10	11	6	0	4
Voriconazole	0	10	3	2	10	14	25	9	3	4	NE	NE	NE
Amphotericin B	NE	0	0	0	1	6	16	48	4	5	NE	NE	NE

NE: not evaluated;

*For caspofungin (CSF), no clinical breakpoints are defined by EUCAST; susceptible isolates to anidulafungin were considered susceptible to caspofungin [18].

Susceptibility profiles according to colors: wild type; non-wild type; susceptible; susceptible, increased exposure; resistant.

**Table 2.** Comparison of the prevalence of resistant or non-wild-type *Candida glabrata sensu stricto* clinical isolates across different antifungal compounds based on minimum inhibitory concentrations (Cochran's Q test).

Sample source	AmB	CSP	MCF	ANF	VRC	*p*
All (n = 80)*	9 (11.3)a	0b	0b	1 (1.3)b	7 (8.8)ab	< 0.0001
Urine (n = 54)	6 (11,1)a	0b	0b	1 (2.8)b	4 (7,4)ab	0.0002
Blood (n = 15)	1 (6.7)	0	0	0	2 (13.3)	0.06
Tracheal secretion (n = 6)	1 (16.7)	0	0	0	0	0.31

Values are expressed as number (percentage).

AmB: amphotericin B; ANF: anidulafungin; CSP: caspofungin; MCF: micafungin; VRC: voriconazole.

*Includes isolates from urine, blood, tracheal secretion, intracavitary abscess, renal abscess, bronchoalveolar lavage, biliary drainage aspirate, and surgical incision aspirate. Different lowercase letters indicate statistically significant differences within the same row (*p* ≤ 0.05) according to the Marascuilo post-hoc procedure or equivalent multiple pairwise comparisons; values followed by the same letter or no letter do not differ significantly (*p* > 0.05).

## Discussion

Isolates of *Candida glabrata* have been extensively studied within evaluations of the *Candida* genus, particularly as causative agents of candidemia [22, 23], though cryptic species identification remains infrequent [9-14]. Brazilian studies [3, 6, 24], when compared with a previous report [25], demonstrate an increasing prevalence of *C. glabrata* as a candidemia agent, which was later confirmed by findings from Brazil [26-28] and other countries, including Japan [29], Sweden [30], and the United States [31]. Conversely, a prior decrease in *C. glabrata* prevalence was observed in Chile, from 25.8% (2000-2006) to 10.3% (2007-2013) [32].

Species-level and cryptic species identification within the *Candida* genus hold significant clinical and epidemiological value [3, 24]. MALDI-TOF MS scores ranging from 2.0 to 2.4 indicate high reliability [33, 34]. All clinical isolates in this study were identified as *Candida glabrata sensu stricto*, which is consistent with literature reporting low prevalences of *C. bracarensis* and *C. nivariensis* [35-39]*.* This finding underscores the epidemiological relevance of our local data.

Antifungal susceptibility testing (AST) revealed a higher prevalence of resistance to amphotericin B (AmB) and voriconazole (VRC) compared with echinocandins [ECNs: caspofungin (CSF), micafungin (MCF), anidulafungin 9ANF)] across both the total cohort of 80 isolates and the sub-cohort of 54 urine isolates. Remarkably, all isolates exhibited susceptibility within the “I” category (susceptible, increased exposure) to fluconazole (FLC). This aligns with Tadec et al. [22], who reported an MIC_90_ ≥ 256 µg/mL for FLC in candidemia patients from a French hospital. Our results also corroborated previous findings of 100% CSF susceptibility (based on breakpoints by Arendrup et al. [40]) and typical AmB MICs, with only 3% of isolates exceeding 1.0 mg/L [41]. A recent study of isolates collected from 22 hospitals in São Paulo found 9.7% of cases attributed to *C. glabrata*, with a 2.8% resistance rate to FLC and VRC, which is comparable to our observed 5.0% rate [4].

ECNs share high structural similarity and overlapping antifungal spectra, exhibiting robust fungicidal activity against *Candida* spp., including resistant isolates [42]. In our study, observed ANF MICs were slightly higher than those of CSF and MCF. This differs from the trends reported by Lindberg et al. [30], who noted MIC_90_ values of 0.03 mg/L, 0.06 mg/L, and 0.012 mg/L for CSF, MCF, and ANF, respectively. The lack of correlation between ECN MICs in pairwise 2 × 2 comparisons may reflect serum-induced phenotypic alterations [43]. 

FLC and VRC are structurally similar and showed direct MIC correlation, albeit with significant activity differences against *C. glabrata sensu stricto*. The prevalence of category “I” susceptibility for FLC and wild-type VRC susceptibility is clinically significant, especially given the rising prevalence of this species. *C. glabrata* is a notoriously challenging target for fluconazole therapy; the wild-type population is natively classified into the “I” category or interpreted as resistant when the MIC exceeds 16 mg/L [44]. 

When comparing our isolates with aggregate EUCAST *C. glabrata* reference data [20], the MICs for AmB, ANF, and MCF were higher in our sample, whereas FLC MICs were lower, and VRC MICs remained similar. This trend, observed clearly when MICs were grouped by concentration ranges, indicates a localized micrological shift toward higher baseline resistance thresholds.

Our observed AmB MICs align well with previous studies [25, 26, 30, 45]. However, the FLC MIC_90_ varied substantially across the literature: it was 8 mg/L in our study, compared to 32 mg/L in Colombo et al. [25], 64 mg/L in Colombo et al. [3], and 16 mg/L in both Nucci et al. [24] and Lindberg et al. [30]. Similarly, reported VRC MIC_90_ values range from 0.5 mg/L [24] to 4 mg/L [3]. ECN MIC_90_ values also show marked inter-study variability. This divergence strongly underscores the necessity of routine local antifungal susceptibility testing to guide definitive treatment regimens and monitor resistance emergence.

A recent review [45] deemed our sample size highly adequate, noting that few single-center evaluations have successfully examined more than 80 isolates. While historical studies focused predominantly on FLC, our comprehensive profiling of ECNs, AmB, and VRC provides a valuable updated baseline. Despite regional variations, our findings align with global trends of low ECN resistance and high FLC category I susceptibility. The lower VRC and higher AmB resistance rates in our study warrant careful clinical consideration, especially since AmB serves as a primary alternative for treating FLC-resistant infections.

Antifungal selection must balance MIC_90_ thresholds with systemic pharmacokinetics, ensuring adequate drug concentrations at the site of infection. For urine and blood, the primary sources of our clinical isolates, AmB and VRC are generally unsuitable for localized urinary tract infections due to their low renal excretion profiles, whereas FLC utility is constrained by decreased susceptibility despite achieving high urinary concentrations [46]. Conversely, for systemic candidemia, treatment strategies may effectively include AmB, VRC, MCF, CSF, or ANF, but should exclude FLC monotherapy due to the pervasive category “I” susceptibility profiles. Other patient-specific variables influencing antifungal choice remain beyond the immediate scope of this study.

Despite the depth of our phenotypic analysis, the single-center nature of this investigation represents a potential limitation. However, given the extreme geographic variability of *C. glabrata* prevalence and susceptibility models across different regions [47], these single-center findings remain highly relevant for establishing localized empirical prescribing guidelines and contributing to broader epidemiological surveillance.

## Conclusion

In conclusion, all evaluated clinical isolates were confirmed as *Candida glabrata sensu stricto*, characterized by low rates of resistance to echinocandins, amphotericin B, and voriconazole, alongside a uniform category “I” (susceptible, increased exposure) profile to fluconazole. Comparative analysis with EUCAST reference distributions highlighted a localized mycological shift toward higher baseline thresholds for certain compounds. Ultimately, these findings establish a strong epidemiological baseline and constitute a valuable diagnostic guide for therapeutic monitoring and antimicrobial stewardship within the regional health system.

## Supplementary material

The following online material is available for this article:

Additional file 1. Distribution of 80 clinical isolates of *Candida glabrata* obtained from patients admitted to the State Hospital of Bauru between 2015 and 2022, according to the clinical specimen type (one sample chi-square test).

Additional file 2. Distribution of 80 patients with infections caused by *Candida glabrata* admitted to the State Hospital of Bauru between 2015 and 2022, according to the hospitalization unit (one-sample chi-square test).

Additional file 3. Comparison of minimum inhibitory concentrations (mg/L) of echinocandins and triazole derivatives determined in 80 *Candida glabrata* sensu stricto clinical isolates (Kruskal-Wallis test for echinocandins and Mann-Whitney U test for triazoles).

## Data Availability

All data generated or analyzed during this study are included in this article.
