# Supplementary material for: Candida glabrata species complex in a Brazilian public tertiary hospital: site of infection, identification and antifungal susceptibility profile
Source: J Venom Anim Toxins Incl Trop Dis. 2026 Aug 10;32:e20250098. doi: 10.1590/1678-9199-JVATITD-2025-0098 (PMC13459181; doi:10.1590/1678-9199-JVATITD-2025-0098)
Supplement: Supplementary file 1 [file 1678-9199-jvatitd-32-e20250098-s1.pdf]

J Venom Anim Toxins incl Trop Dis
https://doi.org/10.1590/1678-9199-JVATITD-2025-0098

Supplementary Material to "*Candida glabrata* species complex in a Brazilian public tertiary hospital: site of infection, identification and antifungal susceptibility profile"

Additional file 1. Distribution of 80 clinical isolates of *Candida glabrata* obtained from patients admitted to the State Hospital of Bauru between 2015 and 2022, according to the clinical specimen type (one sample chi-square test).

Clinical specimen	Patients (number)	Patients (%)
Urine	54A	67.50
Blood	15B	18.75
Tracheal secretion	6C	7.50
Intracavitary abscess	1C	1.25
Renal abscess	1C	1.25
Bronchoalveolar lavage	1C	1.25
Biliary drainage aspirate	1C	1.25
Surgical incision aspirate	1C	1.25
**Total**	**80**	**100.00**

One-sample chi-square test performed using the first three specimens and the sum of the remaining categories: $\chi^2 = 80.1$; $p < 0.001$. Capital letters compare values within the same column; different letters indicate statistically significant differences ($p \leq 0.05$), whereas the same letters indicate no statistical difference ($p > 0.05$).
