# Supplementary material for: Candida glabrata species complex in a Brazilian public tertiary hospital: site of infection, identification and antifungal susceptibility profile
Source: J Venom Anim Toxins Incl Trop Dis. 2026 Aug 10;32:e20250098. doi: 10.1590/1678-9199-JVATITD-2025-0098 (PMC13459181; doi:10.1590/1678-9199-JVATITD-2025-0098)
Supplement: Supplementary file 2 [file 1678-9199-jvatitd-32-e20250098-s2.pdf]

J Venom Anim Toxins incl Trop Dis
https://doi.org/10.1590/1678-9199-JVATITD-2025-0098

Supplementary Material to "*Candida glabrata* species complex in a Brazilian public tertiary hospital: site of infection, identification and antifungal susceptibility profile"

Additional file 2. Distribution of 80 patients with infections caused by *Candida glabrata* admitted to the State Hospital of Bauru between 2015 and 2022, according to the hospitalization unit (one-sample chi-square test).

Hospital unit	Patients (number)	Patients (%)
Wards	30A	37.50
Emergency room intensive care unit (ICU)	12B	15.00
Emergency room	8C	10.00
Adult ICU	9C	11.25
Coronary care unit (CCU)	6C	7.50
Clinical coronary unit	4C	5.00
Burn unit ICU	4C	5.00
Chemotherapy unit	3C	3.75
Ambulatory medical care clinic	1C	1.25
Shelter accommodation unit	2C	2.50
Pediatrics	1C	1.25
**Total**	**80**	**100.00**

ICU: intensive care unit, CCU: coronary care unit. One-sample chi-square test performed using the first five units and the sum of the remaining categories: $\chi^2 = 28.72$; $p<0.001$. Capital letters compare values within the same column; different letters indicate statistically significant differences ($p \leq 0.05$), whereas the same letters indicate no statistical difference ($p > 0.05$).
