# Supplementary material for: Candida glabrata species complex in a Brazilian public tertiary hospital: site of infection, identification and antifungal susceptibility profile
Source: J Venom Anim Toxins Incl Trop Dis. 2026 Aug 10;32:e20250098. doi: 10.1590/1678-9199-JVATITD-2025-0098 (PMC13459181; doi:10.1590/1678-9199-JVATITD-2025-0098)
Supplement: Supplementary file 3 [file 1678-9199-jvatitd-32-e20250098-s3.pdf]

J Venom Anim Toxins incl Trop Dis
https://doi.org/10.1590/1678-9199-JVATITD-2025-0098

Supplementary Material to "*Candida glabrata* species complex in a Brazilian public tertiary hospital: site of infection, identification and antifungal susceptibility profile"

Additional file 3. Comparison of minimum inhibitory concentrations (mg/L) of echinocandins and triazole derivatives determined in 80 *Candida glabrata sensu stricto* clinical isolates (Kruskal-Wallis test for echinocandins and Mann-Whitney U test for triazoles).

Antifungal agent	Median	1st quartile (Q1)	3rd quartile (Q3)	Geometric mean
**Echinocandins**				
Caspofungin	0.03B	0.015	0.03	0.02
Anidulafungin	0.06A	0.03	0.06	0.05
Micafungin	0.03B	0.03	0.03	0.03
p-value	< 0.001			
**Triazoles**				
Voriconazole	0.50B	0.13	0.50	0.26
Fluconazole	1.00A	0.25	4.00	0.89
p-value	< 0.001			

Different letters indicate statistically significant differences within the same column ($p \leq 0.05$); values followed by the same letter do not differ significantly ($p > 0.05$)
